# Assembly and phylogenetic analysis of the complete chloroplast genome sequence of *Actinidia setosa*

**DOI:** 10.1080/23802359.2019.1678423

**Published:** 2019-10-21

**Authors:** Haifeng Lin, Ling Jiang, Fuquan Zhang, Di Bai

**Affiliations:** a College of Information Science and Technology, Nanjing Forestry University, Nanjing, Jiangsu, China;; b School of Civil Engineering, Nanjing Forestry University, Nanjing, Jiangsu, China;; c College of Engineering, Nanjing Agricultural University, Nanjing, Jiangsu, China

**Keywords:** *Actinidia setosa*, chloroplast genome, phylogeny

## Abstract

The complete chloroplast (cp) genome sequence of *Actinidia setosa* (*A. setosa*) was sequenced and assembled into a circular genome of 156,728 bp length using Illumina paired-end data. The cp genome composed of a pair of 24,090 bp inverted repeat (IR) regions separated by a large single copy region (LSC) of 88,256 bp and a small single copy region (SSC) of 20,292 bp. Additionally, a total of 131 genes, including 85 protein-coding genes, 38 tRNA genes and 8 rRNA genes, were identified in the *A. setosa* cp genome. Phylogenetic analysis showed that *A. setosa* was evolutionarily close to other two kiwifruits *A. deliciosa* and *A. chinensis*.

Kiwifruit (genus *Actinidia*), also known as Chinese gooseberry, consisted of more than 70 species, which mainly distributed in East and Southeast Asia from Siberia to Sumatra (Chou et al. 2008; Hsieh et al. 2011). Kiwifruit is particularly rich in vitamin C and vitamin K, and having been given as medicine in traditional Chinese (Tang et al. 2019). Although kiwifruit enjoys worldwide popularity, its ambiguous genetic diversity and genetic differentiation have resulted in long-standing confusion of developing optimum conservation and management strategies. In this study, we report and characterize the complete cp genome sequence of *A. setosa* to provide important genomic resources for promoting its conservation and management.

The leaf of *A. setosa* was collected from South China Botanical Garden, the Chinese Academy of Sciences (Geographic coordinate: 23°11′0.47″N, 113°21′37″E). The genomic DNA was extracted using the DNeasy plant Mini Kit (Quiagen, Carlsbad, CA, USA) and now stored in the Herbarium of South China Botanical Garden (accession number: 20160415AS01), Guangdong, China. Here, we assembled the complete cp genome sequence of *A. setosa* into a free-of-gap genome of 156,728 bp length using AbySS (Simpson et al. 2009), CD-Hit (Li and Godzik 2006), and Minimus2 (Sommer et al. 2007) from Illumina sequencing data, and annotated it using Plastid Genome Annotator (PGA) (Qu et al. 2019). The cp genome sequence was then submitted to GenBank under the accession number of MN326319.

The complete cp genome of *A. setosa* consists of a LSC of 88,256 bp, a SSC of 20,292 bp and a pair of IR regions of 24,090 bp. The overall GC content of the whole cp genome is 37.2%, which is a very common value in *Actinidia*. The GC content of the IR regions was 42.84%, which is higher than that of LSC (35.51%) and SSC (31.18%). According the annotation results, a total of 121 genes were detected in *A. setosa* cp genome, including 85 protein-coding genes, 38 tRNA genes, and 8 rRNA genes. Eight tRNA genes, five protein-coding genes and all four rRNA genes were found to have two copies in the IR regions. Among the total 131 genes, 18 genes were found to contain one intron (7 tRNA genes: *trnK-UUU*, *trnL-UAA*, *trnV-UAC*, *trnI-GAU* × 2, and *trnA-UGC* × 2; 11 protein-coding genes: *rps16*, *rpoC1*, *petB*, *petD*, *rpl16*, *rpl2*, *ndhB* × 2, *rps12*×2, and *ndhA*), while only one gene contains two introns (*ycf3*). In order to clarify the position of *A. setosa* in the phylogenetic tree, we selected other 28 plant cp genomes with 76 conserved protein-coding genes to reconstruct a neighbor-joining (NJ) tree using MEGA6 with 1000 bootstrap replicates (Figure 1). The reconstructed NJ tree strongly support that *A. setosa* was evolutionarily close to other two kiwifruits *A. deliciosa* and *A. chinensis*. The complete cp genome sequence of *A. setosa* will provide important genomic resources to the conservation genetics of this species as well as for the evolution analysis of *Actinidia*.

**Figure 1.**
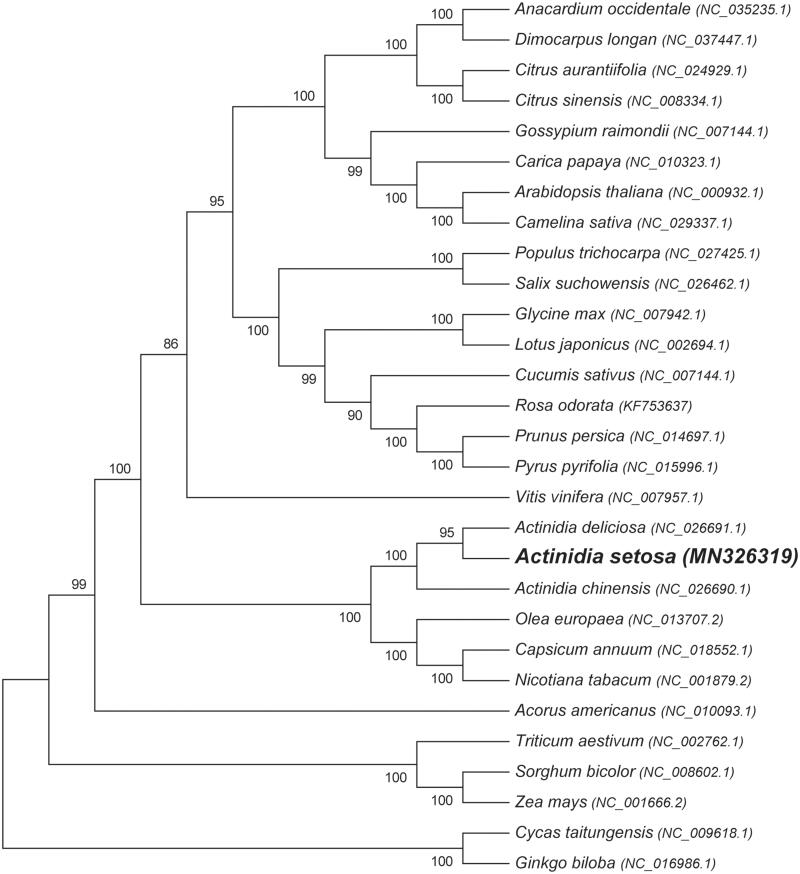
The neighbour-joining phylogenetic tree of 29 plant cp genomes based on 76 conserved genes. Bootstrap values are listed for each node. Accession numbers for tree reconstruction are listed right to their scientific names.
